# 共价三嗪骨架吸附剂-固相萃取-超高效液相色谱-串联质谱法测定牛奶中3种青霉素类残留

**DOI:** 10.3724/SP.J.1123.2022.07002

**Published:** 2022-11-08

**Authors:** Qin LI, Shuyu DAI, Yuan YANG, Yumin FENG, Hongzhen LIAN, Shusheng ZHANG, Wenfen ZHANG

**Affiliations:** 1.郑州市食品药品检验所, 河南 郑州 450006; 1. Food and Drug Inspection Institute of Zhengzhou, Zhengzhou 450006, China; 2.郑州大学化学学院, 河南 郑州 450001; 2. College of Chemistry, Zhengzhou University, Zhengzhou 450001, China; 3.南京大学化学化工学院, 江苏 南京 210023; 3. College of Chemistry and Chemical Engineering, Nanjing University, Nanjing 210023, China; 4.中国烟草总公司郑州烟草研究院, 河南 郑州 450001; 4. Zhengzhou Tobacco Research Institute of China National Tobacco Corporation, Zhengzhou 450001, China

**Keywords:** 共价三嗪骨架, 固相萃取, 超高效液相色谱-串联质谱, 青霉素, 牛奶, covalent triazine frameworks (CTFs), solid-phase extraction (SPE), ultra performance liquid chromatography-tandem mass spectrometry (UPLC-MS/MS), penicillin, milk

## Abstract

建立了固相萃取-超高效液相色谱-串联质谱法(SPE-UPLC-MS/MS)同时测定牛奶中苄青霉素、邻氯青霉素、氨苄青霉素3种青霉素残留的分析方法。以自制的共价三嗪骨架(CTFs)材料作为固相萃取吸附剂,对影响固相萃取柱效率的吸附剂填充量、洗脱剂种类和用量及上样速率等主要因素进行了优化;同时对样品的提取和净化条件进行了考察。在3 mL/min的样品流速下,采用60 mg CTFs吸附剂和6 mL纯乙腈洗涤液达到了最佳的萃取效果。以0.1%甲酸水溶液-乙腈作为流动相进行梯度洗脱,在Waters ACQUITY UPLC BEH C18色谱柱上分离,电喷雾正离子(ESI^+^)模式下以动态多反应监测(MRM)采集数据,外标法定量。3种目标分析物的线性回归方程相关系数均大于0.999,检出限(LOD)为0.05~0.10 μg/kg(信噪比*S/N*=3),定量限(LOQ)为0.1~0.4 μg/kg(*S/N*=10),加标回收率为84.9%~94.1%,相对标准偏差(RSD, *n*=5)为1.66%~3.27%。此外,共价三嗪骨架材料与目标物的作用机理研究表明,主客体分子间存在*π-π*相互作用和氢键作用等多重相互作用,使该吸附剂可成功用于牛奶中青霉素的富集和净化。该方法具有精密度较高、重复性较好、分离度高、分析时间短等优点,可适用于牛奶中青霉素定性定量测定。

## Abstract (en)

A method based on solid-phase extraction-ultra performance liquid chromatography-tandem mass spectrometry (SPE-UPLC-MS/MS) was established for the determination of gpenicillin, cloxacillin, ampicillin residues in milk. Using self-made covalent triazine frameworks (CTFs) as the solid-phase extraction sorbents, the main factors influencing the efficiency of the solid-phase extraction columns, such as the sorbent amount, eluent type, eluent volume, and flow rate, were optimized. The extraction and purification conditions for the samples were also investigated. The optimal extraction effect was achieved at a flow rate of 3 mL/min with 60 mg CTFs and 6 mL eluent solution (acetonitrile). Separation was carried out on a Waters ACQUITY UPLC BEH C18 column, and 0.1% formic acid aqueous solution-acetonitrile was used as the mobile phases for gradient elution. The filtrate was detected by ultra performance liquid chromatography-tandem mass spectrometry, identified by electrospray ionization (ESI) in the positive mode using multiple reaction monitoring, and quantified using external standards. The calibration curves of the three penicillins showed good linearity and the correlation coefficients of the linear regression equations for the three target analytes were all greater than 0.999. The limits of detection (LODs) and limits of quantification (LOQs) were 0.05-0.10 μg/kg and 0.1-0.4 μg/kg, respectively. The average recoveries of the three analytes were 84.9%-94.1%, and the relative standard deviations (RSDs, *n*=5) were 1.66%-3.27%. Moreover, the mechanism of interaction between the CTFs and the target analytes was analyzed. The results revealed the existence of *π-π* and hydrogen-bond interactions between the CTFs and analytes. The results further indicated that the CTFs could be successfully used for the enrichment and purification of penicillins in milk. The proposed method has the advantages of high precision, good reproducibility, high resolution, and short analysis time, and it is suitable for the qualitative and quantitative determination of trace targets in complex matrices.

青霉素类抗生素是*β*-内酰胺类中一大类抗生素的总称,青霉素因高效低毒、成本低,常作为治疗牛乳腺炎和其他细菌感染性疾病的首选药物^[1,2]^。在我国养殖行业滥用青霉素的现象比较严重,甚至为了防止牛奶变坏,一些不法分子会将青霉素添加在鲜奶中以增加保质期,这就给食品安全带来了很大隐患,同时对人们的身体健康也构成了潜在的危害^[3]^。国家市场监督管理总局将乳制品中苄青霉素(gpenicillin)、邻氯青霉素(cloxacillin)、氨苄青霉素(ampicillin)等列入高风险监测项目。因而研究一种准确、快速检测牛奶中青霉素残留量的方法具有重大现实意义。

目前动物源性食品中青霉素残留量的测定方法有很多种,如胶体金免疫层析技术^[4]^、酶联免疫法^[5,6]^、微生物法^[7,8]^、流动注射化学发光法^[9]^、电化学法^[10]^、液相色谱法^[11⇓⇓-14]^和液相色谱-串联质谱法^[15⇓⇓⇓⇓⇓⇓⇓⇓⇓⇓⇓-27]^等多种方法,其中液相色谱-串联质谱法因灵敏度高、适用范围较宽、易确证而被越来越多地应用于残留分析中。

本实验基于自制的共价三嗪骨架材料(CTFs)固相萃取小柱^[28]^来代替商品化固相萃取小柱,通过CTFs与目标物之间的*π-π*、氢键等相互作用力考察该材料富集和检测牛奶中青霉素的可行性。选择苄青霉素、邻氯青霉素、氨苄青霉素3种青霉素化合物作为目标化合物,研究建立一种测定牛奶中青霉素的固相萃取-超高效液相色谱-串联质谱(SPE-UPLC-MS/MS)新方法。

## 1 实验部分

### 1.1 仪器、试剂与材料

Waters Xevo TQ-S超高效液相色谱-串联质谱联用仪(美国Waters公司); TG16-WS台式高速离心机(湖南湘仪实验室仪器开发有限公司); Rocket快速溶剂蒸发仪(英国GeneVac公司); PPM48半自动正压固相萃取仪(美国J2 Scientific公司); Multi Reax全能型振荡器、Heidolph Multi Reax振荡器(德国Heidolph公司); BF2000氮气吹干仪(北京八方世纪科技有限公司); MS204S精密电子天平(瑞士METTLER TOLEDO公司)。

苄青霉素、邻氯青霉素、氨苄青霉素标准样品(德国Dr. Ehrenstorfer公司,纯度>99.9%);乙腈、甲醇均为色谱级(德国默克公司),娃哈哈纯净水(杭州娃哈哈有限公司),甲酸(分析纯,山东鑫博化工有限公司)。

自制共价三嗪骨架材料固相萃取小柱;牛奶样品购自不同超市。

### 1.2 标准溶液的配制

分别精确称取苄青霉素、邻氯青霉素、氨苄青霉素标准品,用乙腈-水(30:70, v/v)溶解并定容至100 mL,配成质量浓度为100 μg/mL的标准储备溶液。

分别取苄青霉素、邻氯青霉素上述标准储备溶液1 mL和氨苄青霉素标准储备液2 mL于100 mL容量瓶中,用0.1%甲酸水溶液定容,配制成混合标准中间储备液。

再分别取0.1、0.2、0.4、0.8、1.0、2.0、5.0 mL混合标准中间液于10 mL容量瓶中,用0.1%甲酸水溶液定容,配制成系列混合标准工作溶液。

### 1.3 样品前处理

准确称取10 g牛奶样品于50 mL离心管中,加入20 mL乙腈-水(15:2, v/v)振荡混匀3 min, 以6000 r/min离心5 min后,将上清液转移至蒸发瓶内;再向离心管中加入10 mL乙腈-水(15:2, v/v),将沉淀捣碎,振荡1 min, 以6000 r/min离心5 min后,合并两次上清液,置于快速溶剂蒸发仪于45 ℃蒸发除去乙腈,之后立即用乙酸盐缓冲液(pH 4.5)溶解,待净化。

将装有60 mg CTFs吸附剂的固相萃取小柱依次用3 mL甲醇、1 mL水进行活化后,将上述待净化液以3 mL/min速率上样,先用1 mL超纯水淋洗,再用6 mL乙腈洗脱,将洗脱液于37 ℃氮气吹干,用0.1%甲酸水溶液定容至1 mL,过0.45 μm有机滤膜后,立即用UPLC-MS/MS进行测定。

### 1.4 分析条件

#### 1.4.1 色谱条件

色谱柱:Waters ACQUITY UPLC BEH C18(50 mm×2.1 mm, 1.7 μm),流动相A为0.1%甲酸水溶液(乙酸铵溶液调节pH 4.5), B为乙腈,梯度洗脱,洗脱程序见表1,流速0.3 mL/min,柱温30 ℃,进样量10 μL,采集时间5 min。

**表1:** 梯度洗脱程序

Step	Time/min	Flow rate/(mL/min)	φ(A)/%	φ(B)/%
1	0	0.3	90	10
2	0.5	0.3	90	10
3	1.5	0.3	70	30
4	3.0	0.3	60	40
5	5.0	0.3	90	10

A: 0.1% (v/v) formic acid aqueous solution; B: acetonitrile.

#### 1.4.2 质谱条件

电喷雾正离子(ESI^+^)扫描,多反应监测(MRM)模式,毛细管电压0.89 kV,锥孔电压85 V,脱溶剂温度500 ℃,锥孔气流速150 L/h,雾化器流速990 L/h,碰撞气流速0.12 mL/min,驻留时间0.3 s。3种青霉素的定性离子对、定量离子对见表2。

**表2:** 3种青霉素的定性和定量离子对

Analyte	Qualitative ion pairs (m/z)	Quantitative ion pair (m/z)
Gpenicillin	335.1/160.0; 335.1/175.0	335.1/160.0
Cloxacillin	436.0/277.0; 436.0/160.0	436.0/277.0
Ampicillin	350.2/106.0; 350.2/192.1	350.2/106.0

## 2 结果与讨论

### 2.1 作用机理讨论

在我们前期^[28]^的研究中发现,本文所制备的三嗪基共价有机骨架材料具有苯环、含氮原子三嗪环等活性位点,可以与核苷分子之间形成包结作用、*π-π*相互作用和OH…N、NH…O氢键作用、LP…*π*相互作用和静电作用等多重相互作用力。本文所选取的青霉素靶标具有羧基、氨基、亚氨基、硫杂原子、苯环等多个活性位点。因此我们推测,与核苷分析类似,自制的共价三嗪骨架材料主体化合物可与目标物青霉素发生上述相同的多重作用力,使其可作为吸附剂成功用于牛奶中青霉素的富集和净化。

### 2.2 样品提取和净化条件的选择优化

考虑到3种青霉素的性质以及自制固相萃取小柱的特殊性,且为了获得较高的回收率,本实验对样品的提取和净化条件进行了选择优化。考虑到基质的影响,样品提取和净化条件的优化均在空白样品基质中进行,即在2.0 μg/mL添加水平下进行考察。

#### 2.2.1 样品提取条件优化

由于青霉素易溶于水,因此本实验考察了乙酸盐缓冲液(pH 4.5)、超纯水、乙腈-水(15:2, v/v)等提取溶液对青霉素的提取效率。结果发现上述提取溶液对3种青霉素的提取效果均差别不大。因乙腈可以沉淀乳制品中的蛋白质和脂肪,为下一步的净化做好准备,参照GB/T 21315-2007方法,最终选取乙腈-水(15:2, v/v)作为提取溶液。

#### 2.2.2 净化条件优化

将提取溶液旋转蒸干后,本实验分别考察了用乙酸盐缓冲液(pH 4.5)、0.1%甲酸水溶液、超纯水等溶液溶解残渣,以期获得最佳净化条件。结果表明,乙酸盐缓冲液(pH 4.5)不仅能完全溶解残渣,且对3种青霉素的回收率最高。

### 2.3 固相萃取条件优化

为了获得较高的回收率,本实验考察了吸附剂填充量、洗脱剂种类和用量、上样速率等因素对固相萃取效率的影响,以期最大限度地对目标物进行富集净化。考虑到基质的影响,固相萃取的条件优化均在空白样品基质中进行,即在0.5 μg/mL添加水平下进行考察。

#### 2.3.1 CTFs吸附剂填充量

吸附剂的用量是影响固相萃取效果的关键因素之一,用量太多会造成材料的浪费,用量太少则不能完全吸附目标分析物。本实验考察了吸附剂填充量为20、40、60、80、100 mg时,CTFs对目标物的吸附效果。结果如图1a所示,随着吸附剂填充量的增加,目标分析物的回收率均逐渐增加,当吸附剂的填充量达到60 mg以后,回收率基本保持不变。因而本实验选择60 mg作为最佳吸附剂填充量。

**图1:**
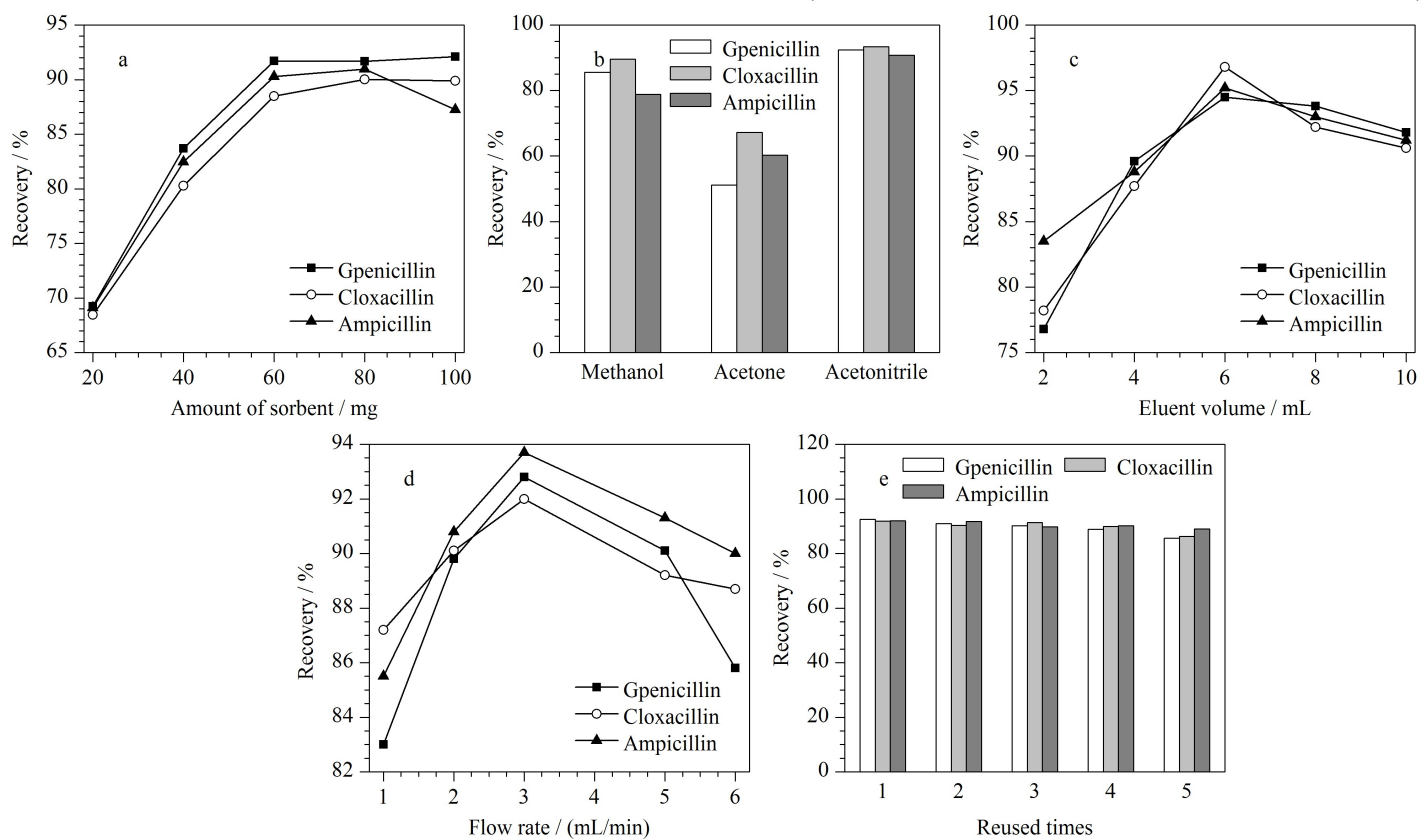
(a)吸附剂填充量、(b)洗脱剂种类、(c)洗脱剂用量、(d)上样速率和(e)吸附剂重复利用次数对3种青霉素回收率的影响

#### 2.3.2 洗脱剂种类

不同的洗脱剂对目标分析物的洗脱能力有差别,为了获得最佳的洗脱效果,本实验对乙腈、丙酮和甲醇3种洗脱剂进行了考察,结果如图1b所示。从图中可以看出,乙腈作为洗脱剂时,3种青霉素的回收率最高,说明乙腈对目标物的洗脱能力最强,因而本实验选用乙腈作为最佳洗脱剂。

#### 2.3.3 洗脱剂用量

洗脱剂的用量对目标物的洗脱程度有直接影响,因此本实验对洗脱剂的用量进行了考察。分别考察了2、4、6、8和10 mL乙腈对3种青霉素的洗脱效果,结果如图1c所示。随着洗脱剂用量的增加,青霉素的回收率也随之升高,当洗脱剂的用量增加至6 mL时,3种青霉素回收率达到最大;继续增加洗脱剂的用量,回收率明显降低,这可能是由于氮吹时间长引起较大损失所致。因此本实验选用6 mL乙腈作为最佳洗脱剂用量。

#### 2.3.4 上样速率

为了在相对较短的时间内获得较高的萃取效率,本实验考察了上样速率对3种青霉素萃取效率的影响,结果如图1d所示。将样品溶液分别以1、2、3、5和6 mL/min的上样速率通过自制SPE小柱,在上样速率为3 mL/min时,3种青霉素回收率达到最大;继续增大上样速率,青霉素与萃取填料的接触时间变短,萃取效率有所下降。因此,本实验选用3 mL/min的速率上样。

#### 2.3.5 吸附材料的重复利用性

吸附剂能否重复多次利用是衡量吸附剂性能的一个重要因素。为了降低成本且保证实验的准确性,期望吸附剂能够重复多次利用。在本实验中考察了吸附剂重复使用次数对回收率的影响,结果如图1e所示。自制SPE小柱经洗涤且重复使用5次后,发现其对目标物仍具有较高的回收率,说明该材料具有较好的重复利用性。考虑到实验样品基质的复杂性以及实验的准确性,在实验过程中每个小柱重复使用2次。

### 2.4 方法学考察

#### 2.4.1 标准曲线及相关系数

取1.2节中的混合标准工作溶液进行二级质谱扫描,获得碎片离子信息,分别以3种青霉素的响应值为纵坐标,标准溶液质量浓度为横坐标,绘制标准曲线,计算出3种青霉素的检出限(LOD)为0.05~0.10 μg/kg(*S/N*=3),定量限(LOQ)为0.1~0.4 μg/kg(*S/N*=10)。从表3可以看出,3种青霉素在各自的线性范围内表现出良好的线性关系,相关系数(*R*^2^)均大于0.999。

**表3:** 3种青霉素的线性方程、相关系数、线性范围和检出限

Analyte	Linear equation	R^2^	Linear range/ (μg/L)	LOD/ (μg/kg)
Gpenicillin	y=8132.91x+2552.59	0.9998	1-50	0.05
Cloxacillin	y=3677.87x-187.575	0.9992	1-50	0.05
Ampicillin	y=37920.7x+40139.8	0.9992	2-100	0.10

*y*: peak area; *x*: mass concentration, μg/L.

#### 2.4.2 回收率和精密度

取均质后的空白牛奶试样,分别加入低、中、高3个不同水平的3种青霉素标准溶液进行加标回收试验,每个加标水平做5次平行试验,得到平均回收率及相对标准偏差,结果见表4。3种目标物的平均加标回收率为84.9%~94.1%,相对标准偏差(RSD, *n*=5)为1.66%~3.27%。结果表明,本方法加标回收率较高,精密度良好,可以满足牛奶样品中青霉素残留量的检测需求。

**表4:** 牛奶中3种青霉素的加标回收率和相对标准偏差(*n*=5)

Analyte	Spiked level/(μg/L)	Recovery/%	RSD/%
Gpenicillin	1	84.9	2.25
	20	89.6	1.94
	50	92.8	3.01
Cloxacillin	1	88.2	2.88
	20	90.7	2.59
	50	91.9	2.38
Ampicillin	2	89.5	1.66
	40	90.6	2.94
	100	94.1	3.27

### 2.5 方法比对

为了考察本方法的有效性,将其与参考文献和国标GB/T 21315-2007、GB/T 22975-2008进行了比较,结果见表5。通过对方法的前处理技术、检测手段、检出限和平均回收率比较显示,本方法具有较低的检出限和较好的回收率。

**表5:** 本方法与相关文献中的方法比较

Analyte	Sample^*^ preparation	Technique	LOQ/ (μg/kg)	Recovery/ %	Ref.
Gpenicillin	LLE	UPLC-MS/MS	5.0	91.1-96.9	[16]
Gpenicillin	SPE	GC-MS/MS	1.7-3.2	83.0-94.5	[17]
Gpenicillin,	SPE	LC-MS/MS	1-2	73.9-90.0	[18]
cloxacillin,					
ampicillin					
Gpenicillin,	SPE	LC-MS/MS	1-2	70.2-96.5	[19]
cloxacillin,					
ampicillin					
Gpenicillin,	SPE	UPLC-MS/MS	0.1-0.4	84.9-94.1	this
cloxacillin,					work
ampicillin					

* Milk; LLE: liquid-liquid extraction; SPE: solid-phase extraction.

### 2.6 牛奶样品中3种青霉素的测定

利用本文所建方法对40批次牛奶样品中的青霉素残留量进行测定,均未检出青霉素。但是从图2中可以看出,对于加标样品,该方法可以很好地实现青霉素的基线分离,且基本无干扰峰,分析时间较短,只需5 min即可完成一次分析。说明本方法在实际样品检测中可以得到较好的分离分析结果。

**图2:**
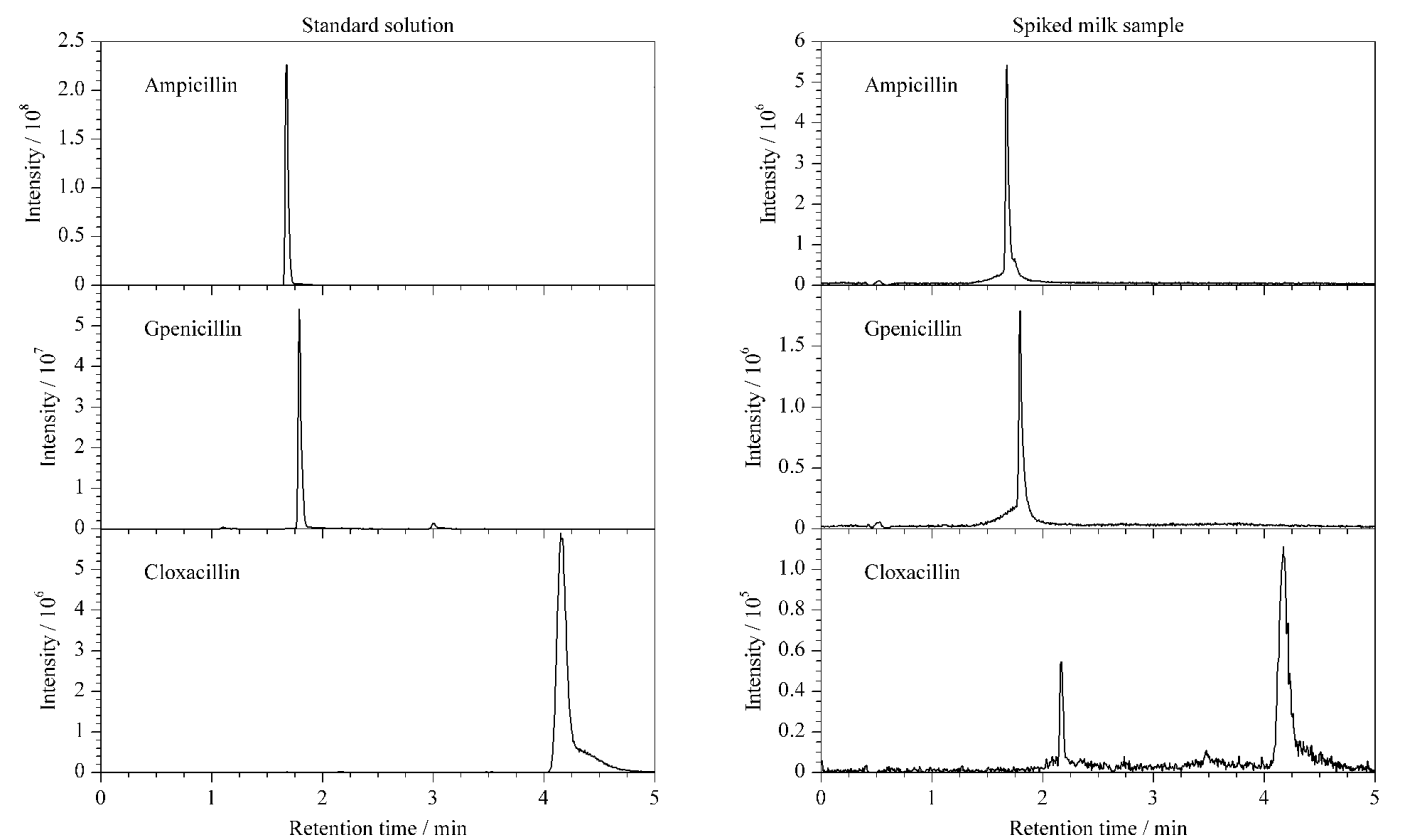
3种青霉素在标准溶液和加标牛奶样品的色谱图

## 3 结论

本文基于自制的CTFs固相萃取吸附剂,对CTFs吸附剂萃取效果进行了考察优化,并获得了最佳条件,建立了同时检测牛奶中3种青霉素类残留量的SPE-UPLC-MS/MS检测方法。所建方法精密度较高,重复性较好,分离度高,实现了对3种青霉素的高效富集,并成功用于牛奶中青霉素类残留量的检测。
